# *Alk7* Depleted Mice Exhibit Prolonged Cardiac Repolarization and Are Predisposed to Ventricular Arrhythmia

**DOI:** 10.1371/journal.pone.0149205

**Published:** 2016-02-16

**Authors:** Shaozhen Ying, Hong Cao, He Hu, Xin Wang, Yanhong Tang, Congxin Huang

**Affiliations:** Department of Cardiology, Renmin Hospital of Wuhan University, Wuhan, China, 238 Jiefang Road, Wuchang District, Wuhan 430060, Hubei Province, China; Georgia State University, UNITED STATES

## Abstract

We aimed to investigate the role of activin receptor-like kinase (ALK7) in regulating cardiac electrophysiology. Here, we showed that *Alk7*^*-/-*^ mice exhibited prolonged QT intervals in telemetry ECG recordings. Furthermore, Langendorff-perfused *Alk7*^*-/-*^ hearts had significantly longer action potential duration (APD) and greater incidence of ventricular arrhythmia (AV) induced by burst pacing. Using whole-cell patch clamp, we found that the densities of repolarizing K^+^ currents I_to_ and I_K1_ were profoundly reduced in *Alk7*^*-/-*^ ventricular cardiomyocytes. Mechanistically, the expression of Kv4.2 (a major subunit of I_to_ carrying channel) and KCHIP2 (a key accessory subunit of I_to_ carrying channel), was markedly decreased in *Alk7*^*-/-*^ hearts. These findings suggest that endogenous expression of ALK7 is necessary to maintain repolarizing K^+^ currents in ventricular cardiomyocytes, and finally prevent action potential prolongation and ventricular arrhythmia.

## Introduction

Arrhythmia is one of the most serious types of cardiovascular disorders, and remains an important cause of mortality and morbidity. Dysregulation of repolarizing potassium current in cardiomyocytes leads to ventricular arrhythmia in humans and animal models [1–6], while the upstream signals that regulate repolarizing potassium currents are poorly understood.

The transforming growth factor beta (TGF-β) superfamily is a large family of structurally related regulatory proteins that function via binding to a distinct group of type I and type II serine/threonine kinase receptors [7]. Binding of TGF-β superfamily members to their type II receptors results in phosphorylation of type I receptors, which subsequently initiates an intracellular downstream signaling cascade in either Smad-dependent or -independent manner [8]. The activin receptor-like kinase 7 (ALK7) is one of the TGF-β type I receptors, and several tissue-specific ALK7 ligands have been reported, including growth differentiation factors (GDFs), nodal, activin B and activin AB [9–12].

The functions of ALK7 have been investigated in a variety of cell across different species. For example, ALK7 negatively regulates glucose-stimulated insulin release in pancreatic β-cell [13] and induces β-cells apoptosis [14]. ALK7 is also abundantly expressed in adipose tissue [15], and endogenous ALK7 is necessary for adipocyte differentiation [16]. ALK7 deleted mice develop age-dependent metabolic syndromes, including progressive hyperinsulinemia, reduced insulin sensitivity, liver steatosis, impaired glucose tolerance and pancreatic island enlargement [13]. Interestingly, *Alk7*^*-/-*^ mice are partially resistant to diet-induced obesity and display reduced fat accumulation [10]. In the cardiovascular system, *Alk7* gene polymorphism has been linked to cardiovascular remodeling in humans [17]. Studies from our group have recently demonstrated that endogenous ALK7 protects against pathological cardiac hypertrophy in mice, suggesting an essential role of ALK7 in cardiovascular remodeling [18].

Structural cardiac remodeling is commonly associated with the disruption of normal cardiac electrical signal and electrophysiology [19, 20]. Given that ALK7 regulates cardiac structural remodeling in the animal model, we hypothesized that ALK7 might also play a role in the modulation of cardiac electrophysiology. In this study, we sought to investigate the role of ALK7 in cardiac electrophysiology remodeling and explore the underlying molecular mechanisms.

## Materials and Methods

### Experimental animals

All animal protocols were implemented under the NIH Guide for the Care and Use of Laboratory Animals (NIH Publication No. 85–23, revised 1996). All the procedures were approved by the Animal Care and Use Committee of Renmin Hospital of Wuhan University. The experimental animals were 10–12 weeks old male *Alk7*^*-/-*^ mice and littermate controls in C57BL/6 background. Mice were provided with food and water, and were maintained in a standard 12-hour light and dark cycle in temperature- and humidity- controlled housing. The physical condition of the animals was monitored once weekly by veterinarians at the institution. Animals were well in general, and no animals died prior to the experimental endpoint. Mice were anesthetized using pentobarbital sodium (60mg/kg, i.p.) to minimize potential suffering during the experiment. The genotyping of *Alk7*^*-/-*^ vs. littermate control mice was assessed by PCR [18].

### Echocardiography and histological analysis

Echocardiography was performed by Sonos 5500 ultrasound (Philips) with a 15-MHz linear array ultrasound transducer. The LV was assessed in both parasternal long-axis and short-axis views at a frame rate of 120 Hz. End-systole or end-diastole was defined as the phase with the smallest or largest LV area, respectively [21, 22]. LVEDD, LVESD, LVEF and FS were measured from the LV M-mode tracing with a sweep speed of 50 mm/s at the midpapillary muscle level [23]. Hearts were excised, washed with saline solution, fixed with 10% formalin and then cut transversely close to the apex to visualize the left and right ventricles. Serial heart sections (4–5 μm thick) were prepared and stained with Masson trichrome stain for collagen deposition and then visualized by light microscopy [24]. The percentage of light blue stain (collagen) was measured with an Image pro-plus 6.0 software.

### Telemetry ECG recording

Mice were anesthetized using pentobarbital sodium (60mg/kg, i.p.), and were then positioned in an incubator to maintain body temperature at 37°C. Leads were tunneled subcutaneously to the right shoulder and left apex (Lead II). Recording began 24 hours after mice recovered from anesthesia. Lead II ECG characteristics were recorded from conscious, freely moving mice under basal conditions in each animal during 24 hours continuously. The ECG amplifier module consisted of high pass filters (set to 0.05Hz), low pass filters (set to 1 kHz) and a gain selection device (set to1000-fold). Signals were digitized at 1 kHz and continuously recorded using a data acquisition system (DSI, US), and analyzed by P3 software [25].

### Preparation of Langendorff-perfused hearts

Animals were anesthetized with pentobarbital sodium (60 mg/kg, i.p.) and heparinized with heparin sodium (100 U, i.p.). Hearts were quickly isolated, excised and transferred to ice-cold (4°C) HEPES-buffered Tyrode’s solution (mM: NaCl 130; KCl 5.4; CaCl_2_ 1.8; MgCl_2_ 1; Na_2_HPO_4_ 0.3; HEPES 10; glucose 10; pH adjusted to 7.4 with NaOH), bubbled with 95% O_2_-5% CO_2_. Hearts were then rapidly cannulated with a tailor-made 21-gauge cannula in aorta and perfused with 37°C HEPES-buffered Tyrode’s solution at 2–2.5 ml/min using a Langendorff-perfusion system (AD Instruments, Australia). Each heart was perfused for 20 minutes before electrophysiology tests. Hearts that did not recover to the regular spontaneous rhythm or had irreversible myocardial ischemia were discarded [25].

### MAP recording and burst pacing

Monophasic action potential (MAP) was recorded from the left and right ventricular epicardium using a custom-made electrode, a string of two 0.25 mm Teflon-coated silver wires (99.99% in purity) galvanically coupled to chloride to eliminate DC offset. The paired platinum stimulating electrode was positioned on the basal surface of the right ventricle, and delivered regular pacing. Signals were amplified using an amplifier (AD Instruments, Australia) and band pass filtered between 0.3 Hz and 1 kHz. MAP waveforms were analyzed using Lab Chrat7.0 software. To induce arrhythmia, burst pacing (2 ms pulses at 50 Hz, 2 s burst duration) was applied for 3 minutes in both ventricular locations to determine susceptibility to ventricular arrhythmia [25].

### Isolation of ventricular cardiomyocytes

Experimental mice were heparinized (100U, i.p.) and anaesthetized using pentobarbital sodium (60mg/kg, i.p.). The hearts were removed, cannulated and retrogradely perfused with the following solutions in the Langendorff system: (a) 5 minutes perfusion with HEPES-buffered Tyrode’s solution (mM): NaCl 130; KCl 5.4; CaCl_2_ 1.8; MgCl_2_ 1; Na_2_HPO_4_ 0.3; HEPES 10; glucose 10; pH adjusted to 7.4 with NaOH); (b) 5 minutes perfusion with Ca^2+^ -free HEPES-buffered Tyrode’s solution; (c) 15 minutes perfusion with Ca^2+^-free HEPES-buffered Tyrode’s containing 0.6mg/ml collagenase type II (Invitrogen Co. US), 0.1% bovine serum albumin, 20 mM taurine and 30 μM CaCl_2_; and (d) 5 minutes perfusion with KB solution (mM: taurine 10; glutamic acid 70; KCl 25; KH_2_PO_4_ 10; glucose 22; EGTA 0.5; pH adjusted to 7.2 with KOH) to washout the remnant digested solution. Perfusion solutions were maintained at 37°C. At the end of the perfusion, the left ventricular free wall was dissected from the heart and placed in ice-cold KB solution. Cardiomyocytes were separated by pipetting, and the cardiomyocytes suspension was stored in KB solution at 4°C prior to electrophysiology test [25, 26].

### Cellular electrophysiology recording

Whole-cell patch clamp was performed using EPC-9 amplifier (List Instruments, Germany), and data were analyzed with Pulse-pulsefit software interface (Version 8.31, HEKA Co. Germany). All experiments were carried out at room temperature (20–22°C). During the experiment, cardiomyocytes were continuously perfused with the extracellular solution (2mL/min) containing (mM): NaCl 130; KCl 5.4; CaCl_2_ 1; MgCl_2_ 1; Na_2_HPO_4_ 0.3; HEPES 10; glucose 10; (pH adjusted to 7.4 with NaOH). The resistance of the pipettes ranged from 2.5 to 3.5MΩ when filled with pipette solution (mM): K-aspartate 110, KCl 20, NaCl 8, MgC1_2_ 1, CaC1_2_ 1, MgATP 4, EGTA 0.1 and 10 HEPES (pH 7.2 with KOH). Series resistance (Rs) was between 4–8 MΩ, and compensation was applied to reduce Rs by 80–90%. Current signals were filtered at 3 kHz by an 8-pole Bessel filter, digitized at a sampling rate of 1kHz, recorded and analyzed by the Pulse-pulsefit software [25].

K^+^ currents recordings: For transient outward current (I_to_), the current-voltage relationship was elicited by a series of 500ms test potentials (-60mV ~ +60mV, with 10mV increments) and a holding potential of -80mV at a frequency of 0.1 Hz. We applied inactivating prepulse (100ms, -40mV) before each test potential to avoid contamination from rapid sodium currents. 0.3mmol/L CdCl was added to the perfusion solution to avoid contamination from calcium currents. For inward rectified potassium currents (I_K1_), the current-voltage relationship was established using 350ms steady-state depolarizing pulses from -120 mV to -40 mV with 10mV increments and a holding potential of -80mV at a frequency of 0.1 Hz. I_K1_ current amplitude (pA) was calculated after subtracting the background currents in the presence of 100 μM BaCl_2_. Current densities (pA/pF) were obtained by normalizing the current amplitudes (pA) to membrane capacity C_m_ (pF).

Steady-state inactivation for I_to_: The two step voltage-clamp protocol was used to assess steady-state inactivation of I_to_. Each inactivating prepulse (-110mV ~ -10mV, with 10mV increments) for 1000ms, was followed by a fixed test pulse (+30mV) for 1000ms. I_to_ currents recorded at each test potential were normalized to the maximal current recorded (I/I_max_). I/I_max_ was plotted for each inactivating prepulse voltage, and data were best fitted to the Boltzmann curve.

Recovery from inactivation for I_to_: an inactivating pulse (P1 = -80mV) with various time intervals (10ms-500ms) was followed by a 500ms test pulse (P2 = +30 mV). The ratio of the current amplitude at test pulse P2 to the current amplitude at inactivating pulse P1 was plotted as a function of time intervals. Time constant was calculated by data fitted to exponential functions [25].

Action potential and resting potential recording: resting potential and action potentials in isolated ventricular myocytes were measured using the whole-cell current-clamp mode [27].

### Quantitative real-time RT-PCR and Western blotting

Total RNA was extracted from frozen, pulverized mouse cardiac tissue using TRIZol (Invitrogen), and cDNA was synthesized using oligo (dT) primers with the Advantage RT-for-PCR kit (BD Biosciences) [28]. The relative amount of cDNA was measured by real-time PCR using SYBR Green PCR Master Mix (Applied Biosystems), and then normalized to that of the housekeeping gene *Gapdh*. Cardiac tissue was lysed in RIPA lysis buffer. Fifty micrograms of cell lysate were used for SDS/PAGE [29], and proteins were then transferred to an Immobilon-P membrane (Millipore). Membranes were then blotted with indicated primary antibodies (all purchased from Sigma-Aldrich) followed by fluorescent labeled secondary antibody (purchased from Li-Cor) [30]. The blots were scanned with the infrared Li-Cor scanner, and quantification of Western blots was performed using an Odyssey infrared imaging system (Li-Cor Biosciences) [23].

### Statistical analysis

Results are presented as mean±SEM [31]. Comparisons between two groups were performed by unpaired Student t test or nonparametric Mann-Whitney U test using SPSS 16.0. Cross Tabulation (Chi-Square Test) with Fischer’s exact test was used for categorical variables. P<0.05 was considered statistically significant. Patch clamp data were analyzed using Origin 9.0 for nonlinear curve fitting.

## Results

### Normal baseline echocardiogram and cardiac histology in *Alk7*^*-/-*^ mice

At baseline, *Alk7*^*-/-*^ and control mice had comparable heart rates and cardiac functions assessed by echocardiograpy (S1A Fig, S1 Table). The heart-to-body weight ratio was also similar between the two groups, indicating no gross cardiac hypertrophy in *Alk7*^*-/-*^ mice (P>0.05) (S1B Fig). In addition, *Alk7*^*-/-*^ hearts did not show any cellular morphologic abnormality or enhanced fibrosis (S1C Fig). These observations are consistent with our previous findings that *Alk7*^*-/-*^ mice exhibited normal heart function and did not develop spontaneous hypertrophic cardiomyopathy at baseline [18].

### Prolonged QT interval in telemetry ECG recording in *Alk7*^*-/-*^ mice

**Fig 1:**
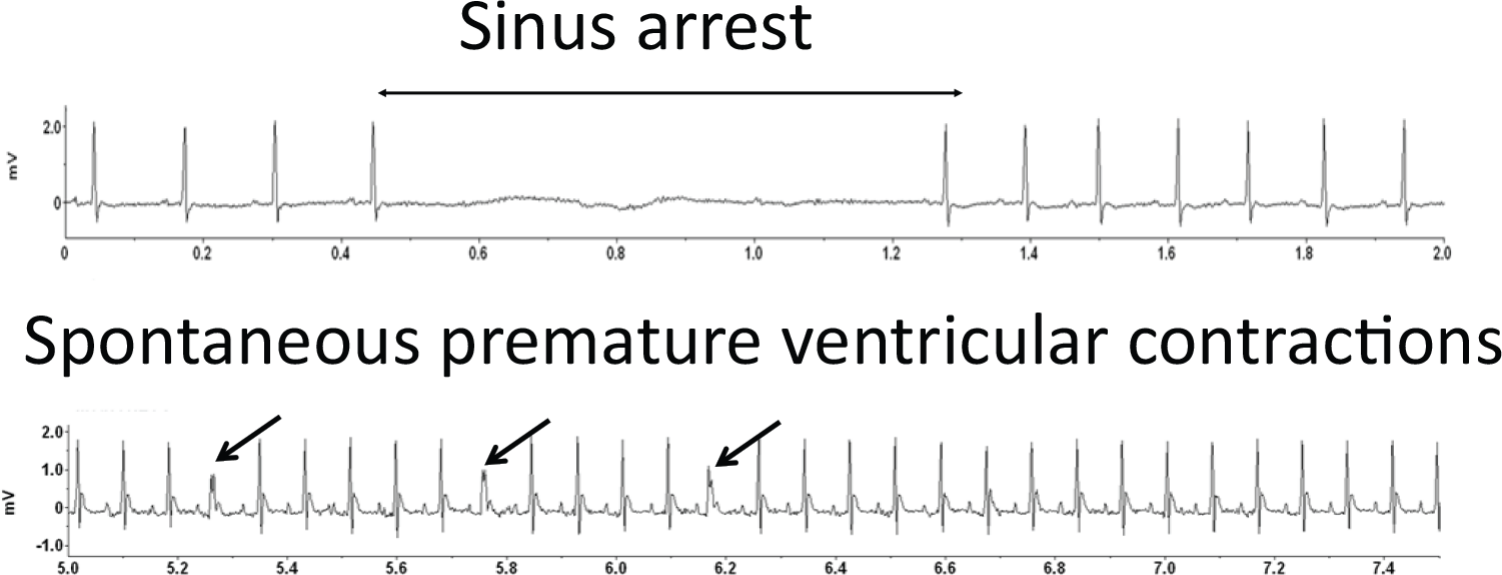
Implantable telemetry Lead II ECG recordings of spontaneous arrhythmia in *Alk7*^*-/-*^ vs. control mice *in vivo*. Sinus arrest (top) and premature ventricular contractions (bottom) were recorded in *Alk7*^*-/-*^ mice.

To explore the function of ALK7 in cardiac electrophysiology, we utilized implantable telemetry ECG to record spontaneous arrhythmia in *Alk7*^*-/-*^ and control mice *in vivo*. During the 24-hour telemetry recording, we captured spontaneous sinus arrest, atrioventricular block and escape rhythm in two out of eight (25%) *Alk7*^*-/-*^ mice, whereas none of the control animals displayed spontaneous arrhythmia (Fig 1). One out of eight (12.5%) *Alk7*^*-/-*^ mice showed sustained premature ventricular contractions (PVC >15 events/min). Notably, QT interval and corrected QT interval (QTc) were significantly prolonged in *Alk7*^*-/-*^ mice by 50% (P<0.05), while the RR, PR or QRS duration were comparable between the two groups (Table 1). Taken together, *Alk7*^*-/-*^ mice exhibited significantly prolonged QT interval in telemetry ECG recording *in vivo*.

**Table 1:** Measurement of ECG parameters in *Alk7*^*-/-*^ vs. control mice.

	Control (n = 10)	Alk7^-/-^ (n = 8)	P value
HR (bpm)	604.9±49.4	589.0±41.9	NS
RR interval (ms)	100.5±8.5	102.4±7.5	NS
PR interval (ms)	34.4±1.8	35.9±2.6	NS
QRS duration (ms)	8.1±0.3	7.8±0.5	NS
QT interval (ms)	43.4±6.0	63.4±5.9	< 0.05
QTc	137.7±18.0	190.7±28.4	< 0.05

Data are presented as mean ± SEM; NS, no statistical significance; Corrected QT Interval (QTc) = QT / $\sqrt{RR/100}$

### Prolonged action potential duration (APD) and increased susceptibility to arrhythmia in Langendorff-perfused Alk7^-/-^ hearts

**Fig 2:**
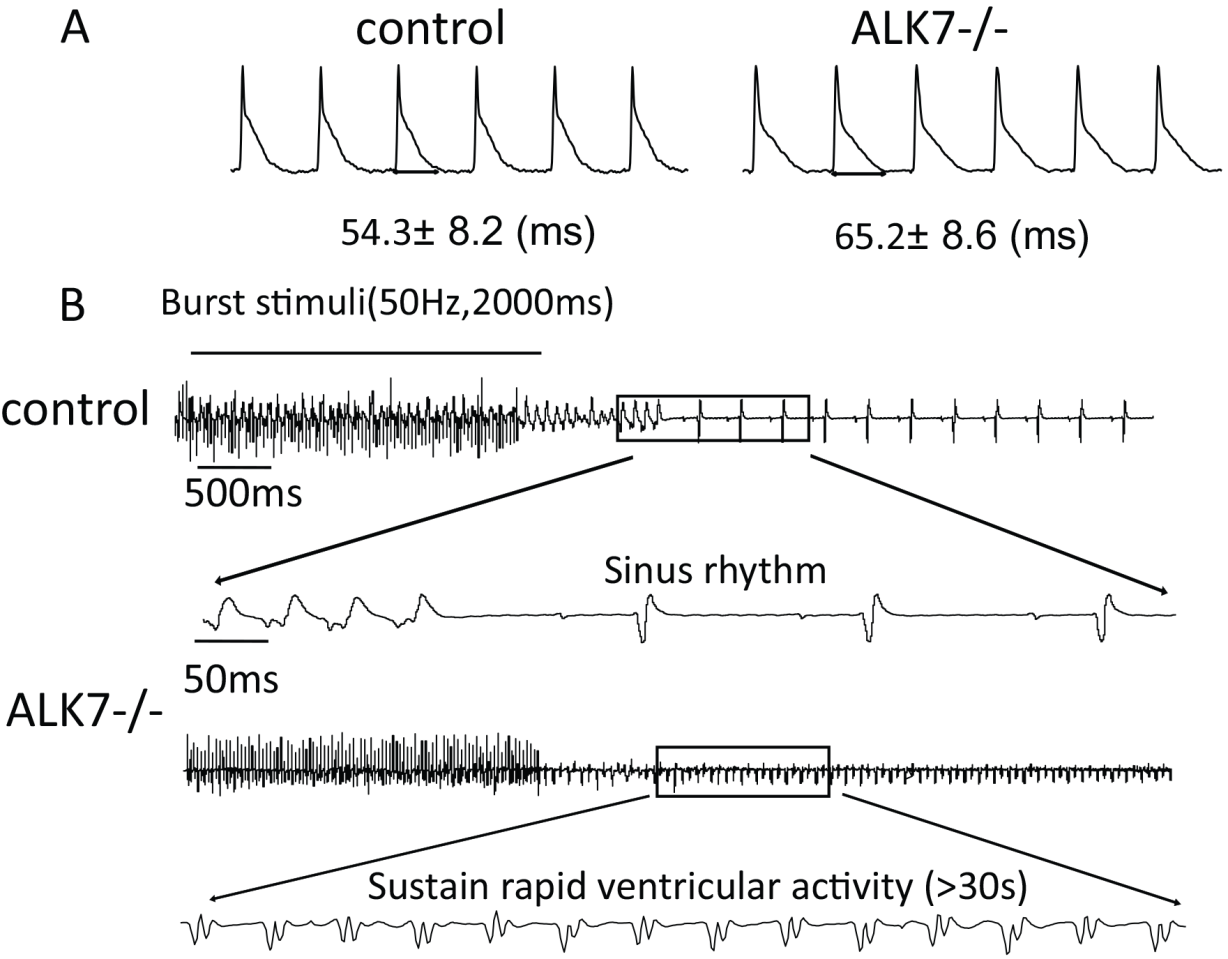
Monophasic action potential (MAP) and ECG recordings of Langendorff-perfused hearts from *Alk7*^*-/-*^ vs. control mice. (A) Representative MAPs at a paced cycle length (CL) of 125 ms, with 22 mV MAP amplitudes. (B) Burst pacing (50 Hz, 2000 ms) was applied to induce ventricular arrhythmias in the Langendorff-perfused hearts from *Alk7*^*-/-*^ and control mice. Shown were representative ECG recordings of transient rapid ventricular activities (duration ≤ 30 s) from the control hearts, and sustained rapid ventricular activities (duration > 30 s) from *Alk7*^*-/-*^ hearts.

We further utilized the Langendorff-perfused heart system to characterize action potential duration (APD) and ventricular repolarization in two groups of mice. Langendorff-perfused hearts were paced at 125 ms cycle length, and action potential duration (APD) at different percentages of repolarization (APD30, 50, 70 and 90) were quantified in Table 2. We observed that the late-phase repolarization duration (APD70 and APD90) were markedly prolonged in the left ventricles of *Alk7*^*-/-*^ hearts (Fig 2A and Table 2). Because prolonged APD enhanced susceptibility to arrhythmia, we predicted that the incidence of arrhythmia induced by burst pacing would be increased in Langendorff-perfused *Alk7*^*-/-*^ hearts. Indeed, the overall incidence of ventricular arrhythmia (VA) was markedly increased in *Alk7*^*-/-*^ hearts (52.0% in *Alk7*^*-/-*^ vs. 19.2% in controls) (Fig 2B and Table 2.). Remarkably, burst pacing-triggered sustained VA (duration > 30s) was recorded in five out of twenty-five (20%) *Alk7*^*-/-*^ hearts but not in any control hearts (Table 3).

**Table 2:** APD in ventricles of Langendorff-perfused hearts.

	Control (n = 13)		Alk7^-/-^(n = 13)	
	LV	RV	LV	RV
APD30	9.3±2.7	9.4±1.7	9.7±2.4	10.1±2.7
APD50	14.1±3.4	13.5±3.3	17.2±4.1	16.8±4.3
APD70	34.4±7.6	33.1±7.8	42.2±10.4*	39.9±11.5
APD90	54.2±8.3	52.6±9.5	65.2±8.6 *	59.6±12.4

Data are presented as mean ± SEM

*P<0.05

APD, action potential duration; LV, left ventricle; RV, right ventricle.

**Table 3:** Incidence of VA by burst-pacing in Langendorff-perfused hearts.

	Control (n = 26)	Alk7^-/-^ (n = 25)	P value
Incidence of VA	19.2% (5/26)	52.0% (13/25)	0.02
Duration of VA			
<10s	15.4% (4/26)	20.0% (5/25)	<0.01
10-30s	3.8% (1/26)	12.0% (3/25)	
>30s	0	20.0% (5/25)	

VA: ventricular arrhythmia

### Decreased outward K^+^ current density in *Alk7*^*-/-*^ ventricular cardiomyocytes

**Fig 3:**
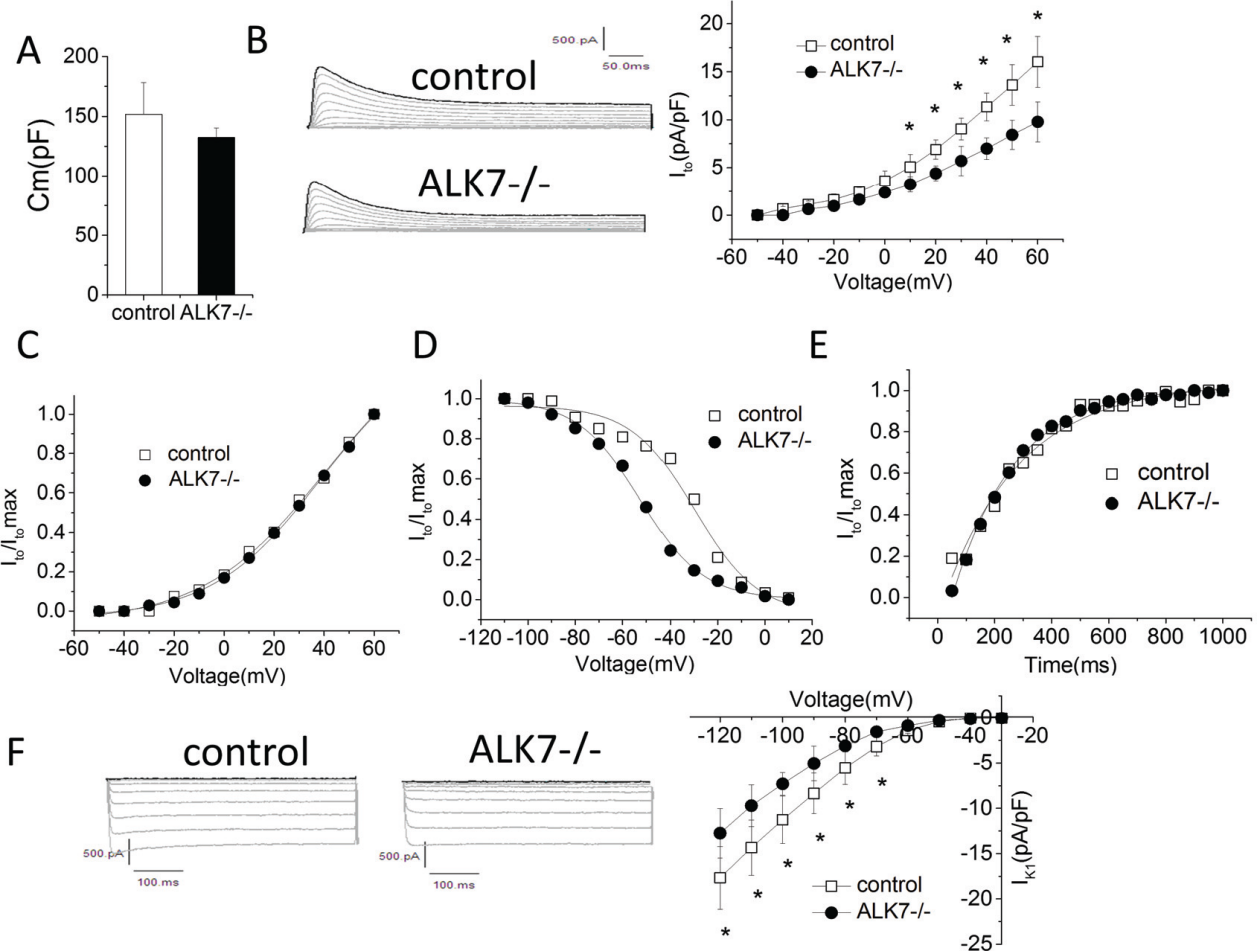
Altered repolarizing K^+^ currents in ventricular cardiomyocytes isolated from *Alk7*^*-/-*^ mice. (A) Membrane capacity (C_m_) was compared between *Alk7*^*-/-*^ and control mice. n = 6 control vs. 6 *Alk7*^*-/-*^animals. (B) Original representative recordings of I_to_ (500 ms depolarization step pulses from—40 mV to + 60 mV with a step size of 10 mV; holding potential (HP) = - 80 mV) (left) and representative current-voltage (I-V) curve for I_to_ in cardiomyocytes from control vs. *Alk7*^*-/-*^ mice (right) (n = 6 control vs. 8 *Alk7*^*-/-*^animals, *P<0.05). (C) Voltage-dependent activation process of I_to_ fitted to Boltzmann distribution curve (I/I_max_) (n = 6 animals /group). (D) Voltage-dependent inactivation process of I_to_ fitted to Boltzmann distribution curve (I/I_max_) (n = 7 animals /group). (E) Recovery following inactivation process of I_to_ fitted to Boltzmann distribution curve (I/I_max_) (n = 6 control vs. 4 *Alk7*^*-/*^ animals). (F) Original representative recordings of the inwardly rectifier I_K1_ current (350 ms voltage steps to potentials between -120 mV and—40 mV; HP = - 80 mV) (left) and representative I-V curve for I_K1_ (right). (n = 8 animals /group, *P<0.05).

Because decreased repolarizing K^+^ current density contributed to APD prolongation in humans and animal models [32], we characterized the major repolarizing K^+^ current, transient outward K^+^ current (I_to_), in isolated ventricular cardiomyocytes using whole-cell patch clamp [33, 34]. *Alk7*^*-/-*^ and control cardiomyocytes had comparable mean cell capacitance (n = 6/group) (Fig 3A). I_to_ current was significantly attenuated in *Alk7*^*-/-*^ cardiomyocytes (16.0±2.6 pA/pF for control vs. 9.8±2.1 pA/pF for *Alk7*^*-/-*^ at +60 mV test pulse; n = 6 vs. 8, P<0.01) (Fig 3B), while half-activated voltage (V_1/2_) was similar (41.0±8.0 mV for controls vs. 35.8±7.9 mV for *Alk7*^*-/-*^, n = 6 vs. 7, P > 0.05) (Fig 3C). Interestingly, *Alk7*^*-/-*^ cardiomyocytes displayed a ~14mV left shift in steady-state inactivation curve (V_1/2_ = -33.6±7.2 for controls vs. -47.7±8.6 mV for *Alk7*^*-/-*^, n = 7/group, P<0.05) (Fig 3D), while the recovery after inactivation of I_to_ was comparable between two groups of cardiomyocytes (n = 6 vs. 4, P>0.05) (Fig 3E).

Inwardly rectified K^+^ current (I_K1_) contributes significant repolarizing current during the terminal phase of repolarization. Reduction of I_K1_ is sufficient to prolong the cardiac action potential and QT interval in humans [1, 35]. We observed that the density of inwardly I_K1_ current was significantly attenuated at voltages ranging from -70 mV to -120 mV (n = 8/group; P<0.01) (Fig 3F). I_K1_ also serves as the primary conductance controlling the diastolic resting membrane potential (Em) in ventricular myocytes [36]. We therefore measured resting Em in two groups of isolated ventricular myocytes and found that the resting Em were comparable (83±9.1 mV for control and 81±7.3 mV for *Alk7*^*-/-*^, n = 5/group, P > 0.05) (S2 Fig). Collectively, *Alk7*^*-/-*^ cardiomyocytes exhibited a significant reduction in repolarizing I_to_ and I_K1_, which could be responsible for the prolonged APD in *Alk7*^*-/-*^ hearts.

### Altered expression of voltage-dependent K^+^ channel subunits in *Alk7-/-* hearts

**Fig 4:**
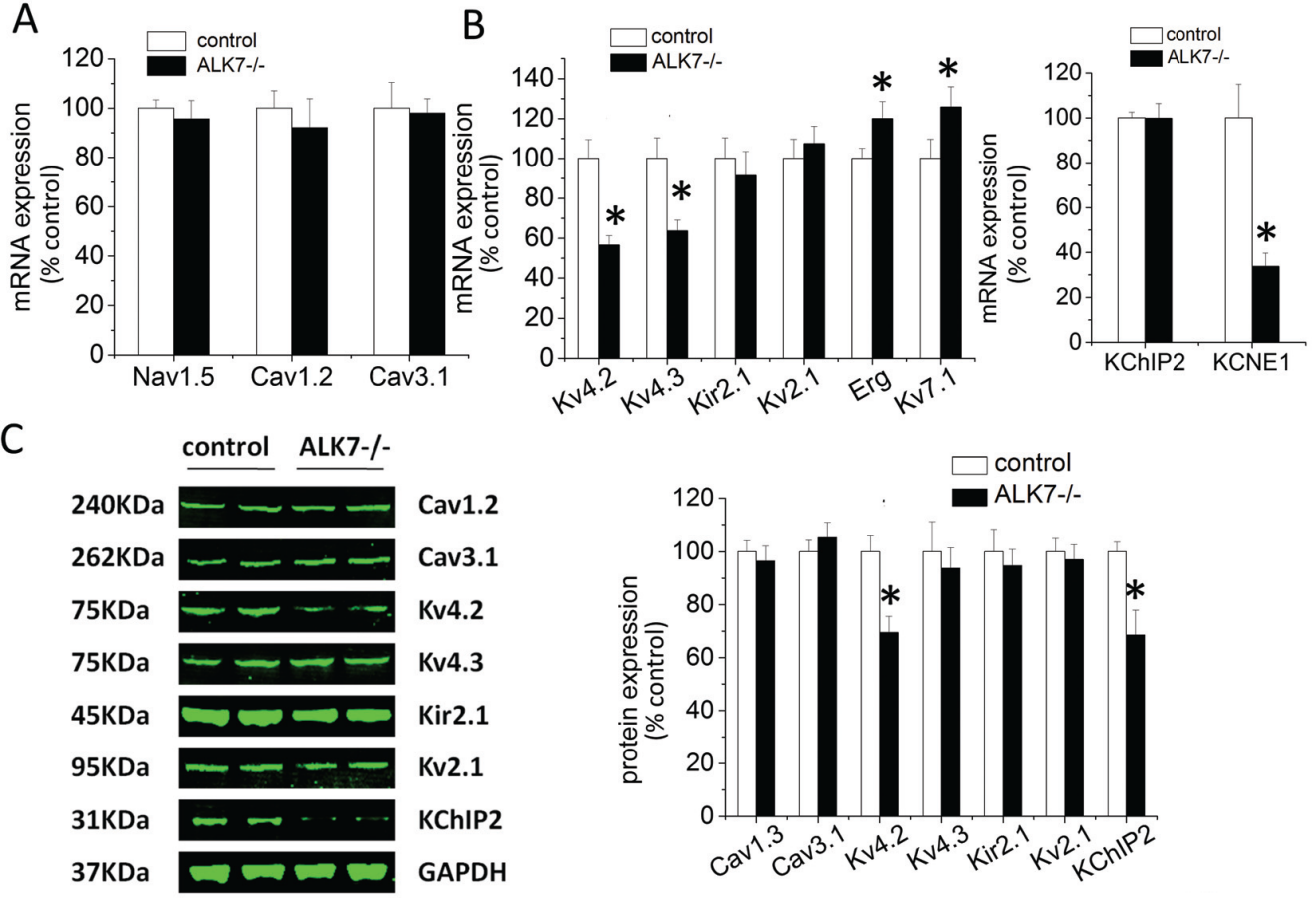
Expression of channels in hearts from *Alk7*^*-/-*^ vs. control mice. (A) mRNA levels of the sodium channel subunit Nav1.5 (I_Na_), the calcium channel subunit Cav1.2 (I_CaL_) and Cav3.1 (I_CaT_). (B) mRNA expression of the potassium channel subunits of transient outward potassium current I_to_ (Kv4.2/4.3), late-phase inwardly rectified K^+^ current I_K1_ (Kir2.1), ultrarapid delayed rectifier current I_Kur_ (Kv2.1), rapid delayed rectifier current I_Kr_ (Erg) and slow delayed rectifier current I_Ks_ (Kv7.1) (left panel). mRNA levels of accessory subunits of I_to_, I_Kr_ and I_Ks_, including KChIP2 and KCNE1 (right panel). n = 4 animals/group, * P < 0.05. (C) Representative western blots of Cav1.2, Cav3.1, Kv4.2, Kv4.3, Kir2.1, Kv2.1 and KChip2 (left) from hearts of two individual animals in each group. Relative abundance of subunits and accessory subunits proteins was quantified and calculated as the % of values in the control mice. n = 4 animals /group, * P<0.05.

We further explored the molecular mechanisms by profiling ion channel gene expression in *Alk7*^*-/-*^ and control hearts. mRNA levels of Nav1.5, Cav 1.2 and Cav1.3, the subunits of sodium and calcium channels, were similar in *Alk7*^*-/-*^ vs. control hearts (Fig 4A). However, mRNA levels of Kv4.2 and Kv4.3, subunits of potassium channel that carried I_to_ current, were markedly reduced in *Alk7*^*-/-*^ hearts by 44.3% and 37.3%, respectively (Fig 4B). Notably, Kv4.2 protein level was also decreased by 30.5% in *Alk7*^*-/-*^ hearts, whereas Kv4.3 protein level was not altered (Fig 4C). K^+^ channel-interacting protein 2 (KCHIP2) is the accessory subunit of Kv4.2 and plays a key role in regulating Kv4.2 functionality and I_to_ current. We found that KCHIP2 protein was reduced by 32.5% but KCHIP2 mRNA was not decreased in *Alk7*^*-/-*^ hearts (Fig 4B right panel and 4C). Thus, the diminished I_to_ current in *Alk7*^*-/-*^ cardiomyocytes could be attributable to the reduction of K^+^ channel subunit Kv4.2 and its accessory subunit KCHIP2.

Rapid delayed rectifier currents (I_Kr_) and slow delayed rectifier currents (I_Ks_) are two major rectifier outward currents during ventricular repolarization [37]. mRNA levels of KCNH2 (Erg) and KCNQ1 (*Kv7*.*1*), the pore-forming subunit for I_Kr_ and I_Ks_ respectively, were moderately increased by 20% in *Alk7*^*-/-*^ mice relative to controls (Fig 4B). Interestingly, mRNA of KCNE, the accessory subunit responsible for both I_Ks_, was significantly reduced by 33.8% in *Alk7*^*-/-*^ hearts (Fig 4C right panel). Kir2.1 is the main subunit of I_K1_ carrying channel. Although the current of I_K1_ was significantly reduced in Alk7 cardiomyocytes (Fig 3F), we did not observe any downregulation of Kir2.1 in *Alk7*^*-/-*^ hearts (Fig 4B left panel and 4C). Taken together, depletion of ALK7 selectively suppressed the expression of Kv4.2, KCHIP2 and KCNE in cardiomyocytes.

## Discussion

Using a genetically modified *Alk7*^*-/-*^ mouse model, we demonstrated for the first time that endogenous ALK7 expression in ventricular cardiomyocyte was necessary to prevent QT interval prolongation, maintain normal APD and protect against ventricular arrhythmia. The arrhythmogenic consequence of ALK7 deletion could be directly mediated by ALK7 signaling, or it could probably be related to adaptive processes after genetic alteration. Thus, to test whether the acute inhibition of ALK7 signaling reproduces the phenotypes of *Alk7*^*-/-*^ mice be helpful to distinguish the two possibilities. Interestingly, *Alk7*^*-/-*^ mice do not have alterations in cardiac histology and cardiac function at baseline, suggesting that ALK7 is either dispensable in maintaining normal cardiac histology/functionality or completely compensated by other pathways.

Prolonged ventricular APD could trigger ventricular tachycardia in humans and in murine models. *Alk7*^*-/-*^ mice exhibited increased QT intervals in telemetry ECG recordings. Furthermore, they had prolonged APD and elevated incidence of ventricular tachycardia in Langendorff-perfused hearts *ex vivo*. Surprisingly, *Alk7*^*-/-*^ mice did not develop spontaneous ventricular *in vivo*. Such discrepancy could probably be explained by the fact that rodents are more resistant to ventricular tachycardia than humans due to the immensely speedy repolarization, small heart sizes and different activation pattern of ventricular myocardium in murine hearts [38]. Because *Alk7* gene polymorphism has been linked to cardiovascular remodeling in humans [17], our findings in murine cardiomyocytes indicate that ALK7 may serve as a potential therapeutic target for ventricular tachycardia in humans. Interestingly, two out of eight (25%) *Alk7*^*-/-*^ mice developed spontaneous sinus arrest, atrioventricular block and escape rhythm, which could lead to bradycardia. This unexpected *in-vivo* finding imply the potential role of ALK7 in pacemaker cells and cardiac electrical conduction system.

Mechanistically, Alk7 deletion in cardiomyocytes resulted in ~40% reduction of I_to_ and a left shift of its steady-state inactivation curve. The decreased I_to_ density could be due to the reduction of Kv4.2 proteins in *Alk7*^*-/-*^ hearts. The left shift of steady-state inactivation could probably be explained by the decreased level of KCHIP2, the accessary subunit responsible for I_to_ inactivation [39, 40]. Notably, the reduced KCHIP2 protein was not associated with a parallel change in KCHIP2 mRNA, suggesting certain posttranscriptional mechanism could modulate KCHIP2 translation or protein stability. Kv4 proteins are critical in maintaining the stability of KCHIP during channel complex trafficking to the membrane and vice versa [41]. Therefore, we speculated that the ~40% reduction in KCHIP2 protein was due to the downregulation of Kv4.2 and subsequent enhancement of KCHIP2 protein instability in *Alk7*^*-/-*^ cardiomyocytes.

Another interesting observation in this study was that the I_K1_ current densities were attenuated in *Alk7*^*-/-*^ cardiomyocytes (Fig 3F), while Kir2.1 mRNA or protein levels were not significantly reduced (Fig 4B left panel and 4C). These results suggest that the decrease of I_K1_ current in *Alk7*^*-/-*^ mice may be due to the post-translational modification of channel properties, and the underlying mechanisms require further investigation.

This study has raised an important question: what are the molecular mechanisms linking ALK7 to potassium channel remodeling and the transcription of Kv4.2 in cardiomyocytes? Several candidate molecular signals have been implicated to regulate the transcription of Kv4.2. For example, activation of AKT and mTOR pathway can enhance Kv4.2 transcription in cerebellar granule neurons and Hela cells [42]. GATA4 in cardiomyocytes also upregulates Kv4.2 transcription [43]. Conversely, calcineurin and NFATc3 signaling suppresses Kv4 expression in the mouse left ventricle during chronic beta adrenergic stimulation [44]. Thus, the expression and function of the abovementioned candidate transcription factors needs to be examined in *Alk7*^*-/-*^ cardiomyocytes. An unbiased transcriptome analysis in control vs. *Alk7*^*-/-*^ cardiomyocytes will provide a more comprehensive survey of differentially expressed genes and further reveal novel functions of ALK7 in cardiomyocytes.

## Conclusion

In the present study, we showed that ALK7 depletion reduced repolarization K^+^ currents, prolonged QT intervals and enhanced the susceptibility to ventricular arrhythmia in mice. The alteration of cardiac electrophysiology in *Alk7*^*-/-*^ mice is not associated with cardiac fibrosis or impaired cardiac function. These findings uncover an unexpected anti-arrhythmogenic role of endogenous ALK7 beyond its role in preventing cardiac fibrosis and structural remodeling. The long QT syndrome may be either congenital or acquired [45]. Given that *Alk7*^*-/-*^ mice exhibit similar phenotypes to that of acquired long-QT syndrome without structural remodeling or heart functions impairment, *Alk7*^*-/-*^ mice are the potentially useful experimental model to study the mechanisms of acquired long-QT syndrome and dysregulated ventricular repolarization.

## Supporting Information

S1 Fig Normal cardiac function in 10–12 weeks old *Alk7*^*-/-*^ mice compared to controls. (A) Representative M-mode echocardiographic images from the control vs. *Alk7*^*-/-*^ mice. (B) Graphs of the ratio of heart weight (HW, mg) to body weight (BW, g) in control vs. *Alk7*^*-/-*^ mice, n = 14/group. (C) Representative Masson trichrome staining of ventricular tissue from control vs. *Alk7*^*-/-*^ mice. Collagen appears as a light blue stain. Bars, 100μm.
(EPS)

S2 Fig Resting and action membrane potential in *Alk7*^*-/-*^ vs. control ventricular myocytes. Representation action potentials of isolated ventricular myocytes from ALK7^-/-^ vs. control mice.
(EPS)

S1 Table Echocardiography parameters in control and Alk7-/- mice. (PDF)
