# Supplementary material for: Alk7 Depleted Mice Exhibit Prolonged Cardiac Repolarization and Are Predisposed to Ventricular Arrhythmia
Source: PLoS One. 2016 Feb 16;11(2):e0149205. doi: 10.1371/journal.pone.0149205 (PMC4755580; doi:10.1371/journal.pone.0149205)
Supplement: S1 Table — (PDF) [file pone.0149205.s003.pdf]

S1 Table. Echocardiography parameters in control and *Alk7*$^{-/-}$ mice

	Control (n=10)	Alk7$^{-/-}$ (n=8)	P value
HR (bpm)	533.5±33.8	563.9±23.4	NS
LVEDD (mm)	3.5±0.2	3.9±0.2	NS
LVESD (mm)	2.0±0.2	2.4±0.2	NS
LVEF (%)	82.0±2.3	75.4±3.4	NS
FS (%)	44.0±2.1	38.3±2.7	NS

Data are presented as mean±SEM.

HR, heart rate; LVEDD, left ventricular end-diastolic diameter; LVESD, left ventricular end-systolic diameter; LVEF, left ventricular ejection fraction; FS, fractional shortening.

NS: no statistical significance (P>0.05).
